# Molecular Insights Into Development and Virulence Determinants of *Aspergilli*: A Proteomic Perspective

**DOI:** 10.3389/fcimb.2018.00180

**Published:** 2018-05-29

**Authors:** Jata Shankar, Shraddha Tiwari, Sonia K. Shishodia, Manali Gangwar, Shanu Hoda, Raman Thakur, Pooja Vijayaraghavan

**Affiliations:** ^1^Genomic Laboratory, Department of Biotechnology and Bioinformatics, Jaypee University of Information Technology, Solan, India; ^2^Amity Institute of Biotechnology, Amity University, Noida, India

**Keywords:** *Aspergilli*, conidia, germinating conidia, proteins, secondary metabolites, proteomics, virulence, virulent factors

## Abstract

*Aspergillus* species are the major cause of health concern worldwide in immunocompromised individuals. Opportunistic *Aspergilli* cause invasive to allergic aspergillosis, whereas non-infectious *Aspergilli* have contributed to understand the biology of eukaryotic organisms and serve as a model organism. Morphotypes of *Aspergilli* such as conidia or mycelia/hyphae helped them to survive in favorable or unfavorable environmental conditions. These morphotypes contribute to virulence, pathogenicity and invasion into hosts by excreting proteins, enzymes or toxins. Morphological transition of *Aspergillus* species has been a critical step to infect host or to colonize on food products. Thus, we reviewed proteins from *Aspergilli* to understand the biological processes, biochemical, and cellular pathways that are involved in transition and morphogenesis. We majorly analyzed proteomic studies on *A. fumigatus, A. flavus, A. terreus*, and *A. niger* to gain insight into mechanisms involved in the transition from conidia to mycelia along with the role of secondary metabolites. Proteome analysis of morphotypes of *Aspergilli* provided information on key biological pathways required to exit conidial dormancy, consortia of virulent factors and mycotoxins during the transition. The application of proteomic approaches has uncovered the biological processes during development as well as intermediates of secondary metabolite biosynthesis pathway. We listed key proteins/ enzymes or toxins at different morphological types of *Aspergillus* that could be applicable in discovery of novel therapeutic targets or metabolite based diagnostic markers.

## Introduction

*Aspergilli* are the ubiquitous and saprophytic fungi that grow on a range of organic materials and help to recycle carbon and nitrogen by decomposing dead organic debris (Mousavi et al., 2016). *Aspergillus* species spread through asexual reproductive bodies called conidia. *Aspergilli* conidia are small in size generally ranging from 2 to 5 μm (Krijgsheld et al., 2013). Due to the small size of conidia they remains suspended in air for a long period of time. As a result, hundreds of these suspended conidia are inhaled by humans and reach to the lower respiratory tract (Latgé, 1999). Conidia are metabolically less active and can remain viable for a long time in the environment (Lamarre et al., 2008). *Aspergill*us genus contain ~250 species that perform diverse biological functions in nature (Krijgsheld et al., 2013). These are one of the most studied filamentous fungi due to their medical (*A. fumigatus, A. terreus, A. niger*) industrial or food importance (*A. oryzae, A. flavus*, and *A. niger;* Krijgsheld et al., 2013; Sugui et al., 2015). Also, it has contributed significantly to the understanding of the biology of eukaryotic organisms and to gain insight into genetics as a model organism (*A. nidulans;* Kniemeyer, 2011). The broad importance of *Aspergilli* pushed it to the forefront of filamentous fungal research among medical to industrial community. The broad morphological and cultural characteristics, significant stress-tolerance chemistry, capability to penetrate host immune system and colonization or damaging the host-tissues and other aspect of its eco-physiology collectively establish *Aspergilli* as a successful pathogen (de Vries et al., 2017). Further, the production of diverse organic acid and commercial enzymes makes them industrially important organisms (Machida et al., 2008; de Vries et al., 2017). Conidia are the main source for the distribution of *Aspergilli* in air or other medium (Latgé, 1999). Immunocompetent hosts are able to efficiently clear these inhaled conidia but in immunocompromised individuals, they persist and often start to germinate to cause a different form of aspergillosis (Thakur et al., 2015). *Aspergillus* infections ranged from invasive aspergillosis to hypersensitive complications. Further, the emergence of drug resistance *Aspergilli* poses a greater threat to human beings (Hagiwara et al., 2016; Sanglard, 2016). In the last few years, efforts have been made to develop novel anti-aspergillus targets or new drug targets to control the infections caused by resistant *Aspergilli* strains (Lamoth et al., 2016; Juvvadi et al., 2017).

The study of *Aspergilli* further increased due to the availability of sequenced genomes of eight *Aspergillus* species (de Vries et al., 2017). This led to the comparative and post genomic studies in *Aspergilli*. Post-genomic studies have raised the questions about the evolution and diversity of work specialization among *Aspergillus* species (Cerqueira et al., 2014; de Vries et al., 2017). Looking at the importance of *Aspergilli* species, proteomic studies were carried out on *A. fumigatus, A. flavus, A. terreus* etc. Due to the morphological features, *Aspergilli* express specific proteins during their transition from resting conidia to mycelium formation, potentially leading to invasion into host tissues and production of secondary metabolites. In recent years, the proteomic studies have enabled scientists to develop new drug/ vaccine and diagnostic targets for various pathogens (Champer et al., 2016; Pérez-Llarena and Bou, 2016). Further, the comparative studies of protein profile at different morphotypes of *Aspergillus* species will help to identify the key cellular processes or biochemical pathways that may be involved in host invasion, stress biology, and production of various secondary metabolites.

**Figure 1:**
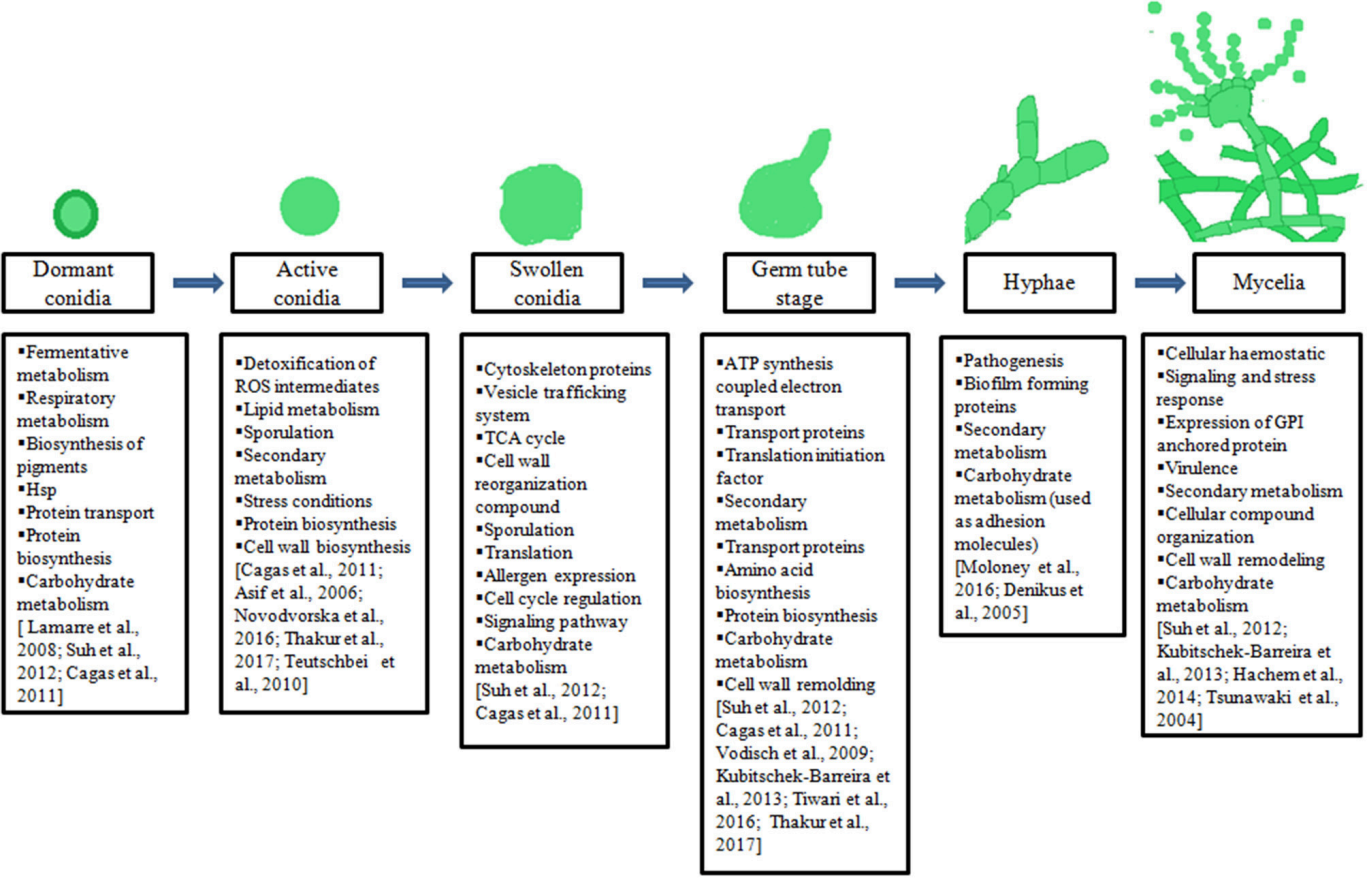
Illustration of different morphotypes of *Aspergilli*, major biological processes underlying during transition of one morphotypes to another mentioned in boxes.

Ascomycetes family produces numerous conidia that are abundantly present in air or environment. These conidia often colonize on food or food products and are involved in the production of various mycotoxins. In addition, they can act as opportunistic pathogens for humans as well as domestic animals. The availability of whole genome of *Aspergilli*, makes it possible to get an insight into the pathogenesis, mycotoxin biosynthesis, or other industrially important metabolites. Furthermore, the comparative genomic studies led to the comprehensive understanding of virulence and morphological variations among *Aspergillus* species. Genetic predictions provide adequate information for targeting subsets of “interesting” biological molecules for further analysis, especially where the presence or absence of a gene can be correlated with a biological function such as, production of industrially important metabolites such as lovastatin by *A. terreus*. The information available through genomic studies should be extended to expression stage at mRNA or protein. The studies at mRNA level in *Aspergilli* will help us to understand the organism's response to external stimuli such as antifungal or mechanism of morphological changes. They also help us to understand the host-pathogen interactions during *in-vitro* or *in-vivo* conditions. However, such studies on mRNA level may not provide the complete picture without relevant studies on functional or protein level. Thus, it is important to study the entire set of proteins expressed at a particular condition. Proteomic studies have been used to understand the complex biological functions, gene expression, and microorganism's interaction with their hosts. In recent years various proteomic studies have been carried out on *Aspergilli* to understand the biology with an aim to develop safe and effective therapeutic agents. Thus, we reviewed the proteins/ enzymes involved in the transition of conidia to mycelial stage and in secondary metabolite biosynthesis in *Aspergilli* species (Figure 1). Further, these studies may help us to carry out comparative proteomic analysis on different *Aspergilli* that may lead to determine common metabolically active processes during the transition from dormant conidia to germinating conidia, as well as to target specific/common therapeutic or diagnostic molecules.

## Determinants to exit conidial dormancy

Asexual spore produced by filamentous fungi including *Aspergilli* (Wyatt et al., 2013) are stress tolerant entities known as conidia, often germinates when provided appropriate conditions such as availability of water and nutrients. It remains in dormancy under unfavorable environmental conditions. Due to the metabolically inactive stage, they can remain viable for a long period of time, sometimes up to 1 year (Lamarre et al., 2008; van Leeuwen et al., 2013). Hence, *Aspergilli* conidia germinates when the conditions are favorable for growth and proliferation (Latgé, 1999). Thus, the transition of conidia to germinating conidia and mycelia are critical for colonization of *Aspergilli* in the suitable host including human cells (Sugui et al., 2015). The adhesion of conidia to hosts cells is an initial step toward germination and maintenance of infections. The remolding of inhaled conidial cell wall initiates their recognition by human soluble and cell bound receptors (Thakur and Shankar, 2016a). If they successfully evade the phagocytic cells, conidia adhere to the respiratory epithelium cells and germinate to form hyphae, thus initiate the invasion into the host tissues (Heinekamp et al., 2015). Proteins that allow exit of conidial dormancy or germination are important for the successful adaptation inside human hosts.

Various proteomics studies on conidia and germinating conidia of *Aspergilli* helped researchers to decipher the mechanism of exiting conidial dormancy and germination. The shot gun proteomic technology provides a detailed insight into the proteins expressed under these morphotypes of *Aspergilli*. Recent studies on *A. fumigatus, A. flavus*, and *A. terreus* are discussed here that demonstrated germination in *Aspergilli* requires set of proteins in conidial stage to exit dormancy or to enter into the isotropic growth followed by germination.

### Protein profile of conidia in *Aspergilli*

Proteome analysis of *Aspergillus* species at different morphotypes has revealed their dynamic nature (Cagas et al., 2011b; Suh et al., 2012). Earlier work on proteins of *A. fumigatus* conidia using 2-DE and MALDI-TOF-MS/MS helped us to gain insight into the germination and pathogenesis of *A. fumigatus* (Asif et al., 2006; Kniemeyer et al., 2006; Vödisch et al., 2009; Kubitschek-Barreira et al., 2013). But this technique has some limitations such as in the detection of low abundant proteins or resolution of large number of proteins. In last few years the shot gun approach has gained importance to carry out proteome analysis of *Aspergilli* at different conditions such as during morphological transitions to stress conditions (in exposure to antifungal or different carbon sources; Suh et al., 2012; Novodvorska et al., 2016; Tiwari et al., 2016; Thakur and Shankar, 2017a). Such studies revealed the broad biological nature of these *Aspergilli* as well as their survival needed in the environment where they face multi-factorial challenges. Cagas et al. explored proteins at different stages of *A. fumigatus* likewise conidia (0 h), swelling conidia (4 h), germinating conidia (8 h), and hyphae (16,h) using iTRAQ technique. A total of 461 proteins were reported during these time points with differential expression patterns. They observed high expression of RodA, hydrophobin protein, Abr2 protein (involve in melanin biosynthesis), heat shock protein hsp30/hsp42, superoxide dismutase SodC, and putative carboxylase in conidia of *A. fumigatus* (Cagas et al., 2011b). Previously, it has been reviewed that heat shock proteins are involved in stress biology and morphogenesis of fungi (Tiwari et al., 2015). Further, Suh et al. also studied the developmental stage specific proteome of *A. fumigatus*. They have carried out the proteome analysis of conidia, isotropic conidia, germ tube, and pre-septation hyphae using a shotgun proteomic approach. They observed 52 enriched proteins in dormant conidia (0 h) that were not observed in other morphological stages (4 h, 8 h, and 16 h). At least half of these proteins have no biologically assigned functions whereas rest of the proteins were involved in stress conditions, cell wall biosynthesis, sporulation, and secondary metabolite biosynthesis pathways. In this study the most abundant protein in dormant conidia was Grg1, however, to attribute biological function to this protein further experimentation is needed.

Other protein identified in dormant conidia of *A. fumigatus* were ConJ, scytalone dehydratase Arp1 (involve in biosynthesis of pigment), and heat shock proteins (Scf1/Awh11, Egd2, Hsp70 chaperone BipA, and calnexin c1xA; Suh et al., 2012). Asif et al. reported conidial surface proteins using 2-DE. They observed major conidial surface proteins such as RodA, PEP2, lipase, putative disulfide isomerase, and fructose−1, 6-bisphosphatase. An allergic protein Aspf3 was also identified from conidial surface (Asif et al., 2006). Teutschbein et al. studied the intracellular proteins of *A. fumigatus* conidia using 2D-PAGE and MALDI-TOF-MS/MS. They reported 57 highly expressed proteins in conidia as compared to mycelia. Most of the identified proteins were involved in pigment biosynthesis, conidial rodlet layer formation and detoxification of reactive oxygen intermediates (Teutschbein et al., 2010). There are limited proteomic studies on other *Aspergilli* conidia. Recently, Thakur and Shankar reported proteins from germinating conidia or conidial proteins of *A. terreus*. A total of 42 conidial proteins were observed and majority of these proteins were from secondary metabolite biosynthesis, lipid metabolism and protein biosynthesis (Thakur and Shankar, 2017a). Recently transcriptomic/translational study by Novodvorska et al. demonstrated that *A. niger* conidia suspended in water have metabolic activity as compared to completely dried conidia. They noticed low level of O_2_ consumption and generation of CO_2_ which suggested that there is a requirement of low respiratory metabolism for conidial maintenance. Their mRNA profiling indicated the presence of fermentative and respiratory transcripts in resting conidia (Novodvorska et al., 2016). Thus, these metabolic pathways were active in conidia. In addition, conidia may require proteins from secondary metabolite pathway and respiratory metabolism for swelling. In conclusion these studies suggested that conidia require a set of proteins to adapt into stress environment to maintain conidial dormancy.

### Proteins associated to swelling conidia of *Aspergilli*

Dormant conidia of *Aspergilli* remains suspended in environment or viable for upto 1 year (Novodvorska et al., 2016). Triggering of conidial germination and isotropic growth requires external stimuli such as contact of conidia with water and nutrients such as glucose, amino acids etc. (Osherov and May, 2000, 2001; Hayer et al., 2013). Till date, fewer studies have been dedicated to identify proteins expressed during conidial swelling of *Aspergilli*. These studies were carried out only on swelling of *A. fumigatus* conidia. Suh et al. reported 215 proteins at 4 h time-point or in expanding conidia of *A. fumigatus*. A total of 85 proteins were highly expressed in swelled conidia in comparison to resting conidia. Most of these proteins were involved in TCA cycle and in protein biosynthesis. The cell wall associated proteins were also observed in expanding or swelling conidia such as Ecm33 and GPI-anchored proteins (beta-1, 3–endoglucanase EglC). The identification of these proteins in swelling conidia suggested cell wall reorganization during isotropic growth. Apart from these proteins, they have also observed the expression of allergen Asp F8/60S ribosomal protein P2, ribosomal subunit proteins and cell cycle regulatory protein Wos2 during expansion of *A. fumigatus* conidia (Suh et al., 2012). Further, Cagas et al. observed the proteins of *A. fumigatus* for early developmental stage using iTRAC technique at 4 h time-point. These proteins were also from protein biosynthesis and TCA cycles in *A. fumigatus* (Cagas et al., 2011b). Thus, these studies suggested that the swelling or expansion of *Aspergillus* conidia requires translation, carbohydrate metabolism, and cell cycle regulation (Figure 1). These biological machineries are necessary for cellular growth or formation of germ tube as well as activation of metabolism in dormant conidia.

### Proteins associated with germinating conidia

The swelling of conidia results in isotropic growth and polarity establishment, leading to the formation of germ tube or germination of *Aspergilli* conidia (Novodvorska et al., 2016). Germination of *Aspergilli* conidia is the key step for the establishment of successful invasion or infection in human host. Knowledge on proteins from germinating conidia of *Aspergilli* will expedite the advancement in diagnosis, vaccine development, and/or drug target identification against *Aspergilli* associated infections (Thakur and Shankar, 2016b; Tiwari et al., 2016). Different studies were carried out on germinating conidia of different *Aspergillus* species. Previous studies on *A. fumigatus* using 2-DE and MALDI-TOF showed higher expression of protein that function in metabolism, protein biosynthesis, transport, and translation initiation factors in germinating conidia as compared to hyphae (Vödisch et al., 2009; Kubitschek-Barreira et al., 2013). Proteins from primary metabolism such as enolase/Aspf22 and glyceraldehyde 3-phosphate dehydrogenase were observed on hyphal surface suggesting that these proteins act as adhesion molecules against host (Denikus et al., 2005; Moloney et al., 2016). Using 2D-DIGE approach, it has been observed that proteins involved in secondary biosynthetic pathways and proteins biosynthesis were over expressed in germinating conidia, while in hyphae, most abundant proteins expressed were from metabolic processes (Kubitschek-Barreira et al., 2013). Cagas et al. observed high expression of RodA a hydrophobin protein, Abr2 protein involve in melanin biosynthesis, heat shock protein hsp30/hsp42, superoxide dismutase SodC, and putative carboxylase in conidia and thereafter decreased in other morphological forms. The higher expressions of proteins that are involved in protein biosynthesis were reported after 4 h of growth. Thus, it suggests that translation is imperative for germination of conidia. Other proteins that were expressed after 4 h of growth were involved in carbohydrate metabolism and respiratory functions (Cagas et al., 2011b). Further, Suh et al. reported 215 proteins in early germ tube and hyphal stage, and out these, 127 were highly expressed in germinating conidia as compared to dormant conidia. Most of these highly expressed proteins were involved in carbohydrate metabolism, protein biosynthesis, amino acid biosynthesis, and ATP synthesis coupled electron transport (Suh et al., 2012). The most abundant proteins expressed during germinations were translation elongation factor subunits, ribosomal components, enzymes from thiazole biosynthesis (Thif and glyceraldehydes 3-phosphate dehydrogenase GpdA) and H^+^-ATPase from plasma membrane, beta-1-3-endoglucanase EglC and Ecm33 (Kumar et al., 2011; Suh et al., 2012). These proteins observed to be involved in cell wall remolding during the germination of conidia.

Tiwari et al. carried out the proteome analysis of germinating conidia of *A. flavus* at 7 h time-point using nano-LC-Q-TOF. A total of 416 proteins have been identified during germination of *A. flavus*. These proteins were associated with carbohydrate and amino acid metabolism along with protein biosynthesis. They also observed several proteins involved in various biological functions such as signaling, transport, and regulation of various cellular processes. Proteins involved in carbohydrate metabolism during germination were enolase, hexokinase and various glucosidases. Other proteins that involve in protein biosynthesis likewise 40S ribosomal protein S1, eukaryotic translation initiation factor-3 subunit-A were among the other proteins expressed during germination. Protein kinase C, mitogen-activated protein kinase MpkC, tyrosine protein phosphatase CdcA and serine threonine protein kinase MARK2 were the expressed proteins during germination of *A. flavus* conidia that function in MAPK signaling pathway (Tiwari et al., 2016). This study suggested that the *A. flavus* conidial germination is accompanied by MAPK signaling pathway. Pechanova et al. identified 538 proteins from mycelia of aflatoxigenic *A. flavus* strain using 2-DE and MALDI-TOF-MS/MS. They have observed the expression of protein that are involved in metabolism of carbohydrate, cellular component organization, cellular haemostatis, signaling, and stress response (Pechanova et al., 2013). The expression of housekeeping enzymes from primary metabolism such as glyceraldehyde-3-phosphate dehydrogenase, ATP synthase beta subunit, enolase, pyruvate decarboxylase PdcA and superoxide dismutase were common between *A. flavus* and *A. fumigatus* at mycelial stage of morphological transition. Furthermore, the expression of GPI anchored proteins such as GPI monnositol transferase 3 and GPI ethanol amine phosphate transferase 1 in germinating conidia of *A. flavus* suggested reorganization of the cell wall during transition phase (Tiwari et al., 2016). Thus, reorganization of the cell wall component occurs during development of conidia into the mycelia/hyphae.

In *A. terreus*, an emerging medically important *Aspergilli* with intrinsic resistance to Amphotericin B (Hachem et al., 2014), Thakur and Shankar identified 373 proteins during the germination of conidia. Most of these proteins were involved in protein biosynthesis and carbohydrate metabolism. Apart from these identified proteins, they also observed the expression of cell wall remolding, virulence and protein that function in secondary metabolites (Thakur and Shankar, 2017a). Previous studies suggested that exit of conidial dormancy is associated with change in fermentative metabolism to respiratory metabolism and protein biosynthesis (Suh et al., 2012; Tiwari et al., 2016). Thakur and Shankar carried out the interactome analysis that predicted proteins from protein biosynthesis, protein transport and carbohydrate metabolism facilitating the exit from conidial dormancy. During germination rRNA processing protein CgrA, hog1, and MpkC were also identified. Thus, their expression during germination of *A. terreus* conidia suggested their involvement in invasion of host tissue as predicted in *A. fumigatus* knockout studies. The expression of arginine biosynthesis bifunctional protein ArgJ in germinating conidia reveled that *A. terreus* conidia may require arginine during germination. Proteins such as 1, 3-β-glucan synthase, chitin synthase, and endopolygalacturonase B were also observed during germination (Thakur and Shankar, 2017a). These proteins indicated their role in cell wall remolding from conidia to germinating conidia in *A. terreus*. Recently, Novodvorska et al. carried out the proteomic study on *A. niger* germinating conidia (at first hour of germinating conidia) and observed the expression of 672 proteins (Novodvorska et al., 2016). The GO analysis of these proteins suggested that these proteins were involved in carbohydrate metabolism, oxidative phosphorylation and protein biosynthesis. The list of common protein expressed during development stages of *Aspergillus* species conidia is given in Table 1.

**Table 1:** List of common proteins that are expressed in germling conidia/ germinating conidia in *Aspergillus* species.

**Name of protein**	***A. fumigatus* (Cagas et al., 2011b; Suh et al., 2012) (8 h)**	***A. flavus* (Tiwari et al., 2016) (7 h)**	***A. terreus* (Thakur and Shankar, 2017a) (16 h)**
**TRANSLATION**			
Eukaryotic translation initiation factor 3 subunit G	+	+	+
40S ribosomal protein S1	+	+	+
40S ribosomal protein S0	+	+	+
Eukaryotic translation initiation factor 3 subunit B	+	+	+
Eukaryotic translation initiation factor 3 subunit C	+	–	+
Eukaryotic translation initiation factor 3 subunit E	+	–	+
**CARBOHYDRATE METABOLISM**			
Endo-beta-1,4-glucanase D	–	+	+
beta-galactosidase B	–	+	+
D-xylulose kinase A	–	+	+
alpha-glucuronidase A	–	+	+
endopolygalacturonase I	–	+	+
Ubiquitin carboxyl-terminal hydrolase CreB	–	+	+
Methylthioribulose-1-phosphate dehydratase	–	+	+
rhamnogalacturonate lyase B	–	+	+
Mannitol-1-phosphate 5-dehydrogenase	–	+	+
**CELL CYCLE**			
Histone H2A	+	–	+
Histone H2B	+	–	+
Nucleolar protein 58	+	–	+
Nuclear distribution protein NudF	+	–	+
Dicer-like protein	–	+	+
endonuclease lcl3	–	+	+
Structure-specific endonuclease subunit Slx1	–	+	+
ATP-dependent RNA helicase Ded1	+	–	+
ATP-dependent RNA helicase Fal1	–	+	+
Methionine aminopeptidase 2-2	–	+	+
Extracellular metalloproteinase Mep	–	+	+
rRNA biogenesis protein Rrp36	–	+	+
**ENERGY AND OTHERS**			
ATP synthase subunit beta	+	–	+
ATP synthase subunit d	+	–	+
Required for respiratory growth protein 9	–	+	+
NADH-cytochrome b5 reductase 2	+	–	+
Nascent polypeptide-associated complex subunit alpha	+	–	+
Polyadenylate-binding protein, cytoplasmic and nuclear	+	–	+
Pyruvate carboxylase	+	+	+
Pyruvate decarboxylase	–	+	+
Catalase-peroxidase	+	+	+
Arginine biosynthesis bi functional protein ArgJ	–	+	+

### Protein profile in hyphae and mycelium

As conidia of *A. fumigatus* expand isotropically upto 4 h at 37°C in complete medium, these cells starts polarizing and send out the first germ tube between 5 and 6 h and continue to elongate to becoming hyphae. The first septum forms near the base of the hyphae between 9 and 10 h, asymmetrically dividing the hyphae into two compartments. At about the same time, first branch forms on the apical side of the septum. Hyphae continue to elongate and branch and eventually form a mycelial mat (Suh et al., 2012). We compiled the data with most abundantly expressed proteins which are hyphal and mycelium specific in *Aspergillus* species. Most of the reports are on *A. fumigatus* in comparison to *A. flavus* and *A. niger*, therefore, we have enlisted 15 hyphal and mycelial proteins found most often in three *Aspergillus* spp. (Table 2). From the GO data it can be inferred that these proteins are involved in cellular metabolism, protein synthesis, transport processes, cell cycle, and virulence (Vödisch et al., 2009; Lu et al., 2010; Pechanova et al., 2013). Proteome map of mycelium of *A. fumigatus* was given by Vödisch et al. using MALDI-TOF-MS/MS showed 381 spots representing 334 proteins. According to their GO annotation, analysis for 416 mycelial proteins, generalizing very detailed categories, 33 different annotation categories were selected and many proteins are found in the categories such as cellular localization, electron transport, metabolic process, cellular metabolic process, and amino acid metabolic process (Vödisch et al., 2009). Further, another proteome set on differential growth conditions were studied by Teutschbein et al. and compared their proteome data with previous reports and observed significant alcohol dehydrogenase activity in resting conidia of *A. fumigatus*, which strongly increased in mycelium grown in ethanol, whereas, low expression and no activity was detectable after stimulation of mycelial growth by glucose. However, using DIGE experiment, cobalamin-independent methionine synthase MetH/D, glutamine synthetase, and thiamine biosynthesis protein were observed to be over expressed in mycelium instead of resting conidia grown on glucose and concluded that enzymes of distinct metabolic pathways are already present in dormant conidia and become less or more abundant during germination and mycelial growth depending on the nature of the environmental stimulus (e.g., the carbon source; Teutschbein et al., 2010). These initial studies shed light on a global view of proteins associated with different morphotypes, which provided a platform to study the transition of one morphotype to another in this fungus.

**Table 2:** List of common proteins that are expressed in hyphae or mycelial morphotypes in *Aspergillus* species.

**Protein**	***A. fumigatus* (Vödisch et al., 2009; Teutschbein et al., 2010; Cagas et al., 2011b; Suh et al., 2012; Kubitschek-Barreira et al., 2013)**	***A. flavus* (Pechanova et al., 2013)**	***A. niger* (Lu et al., 2010)**
Glyceraldehyde 3-phosphate dehydrogenase GpdA	+	+	+
Outer mitochondrial membrane protein porin	+	+	–
Enolase/allergen Asp F 22	+	+	–
ATP synthase F1, b subunit, putative	+	+	–
Allergen Asp F3	+	+	–
Phosphoglycerate kinase PgkA	+	+	–
Cu, Zn superoxide dismutase SOD1	+	+	_+_
Alcohol dehydrogenase	+	+	–
NAD-dependent formate dehydrogenase AciA/Fdh	+	+	–
Putative uncharacterized protein	+	+	–
Cytochrome c subunit	+	+	–
CipC-like antibiotic response protein	+	+	–
GPI-anchored protein	+	+	–
Ran-specific GTPase-activating protein 1, putative	+	+	–
1,3-beta-glucanosyltransferase Bgt	_+_	–	_+_

There are very few reports till the date that attempted to study the temporal protein expression profile in *Aspergillus* spp. Recently, Suh et al. shed light on pre-septation hyphae proteins that are more likely associated with the events after germination. According to their study a total of 119 proteins were upregulated at 8 h of fungal growth in comparison to dormant conidia and 26 proteins were involved in translation as ribosomal subunits/components of a translation elongation factor. They also suggested that telomere and ribosome associated protein Stm1 and glycine-rich RNA-binding protein, a candidate biomarker for hyphal stage in *A. fumigatus* (Suh et al., 2012). Kubitschek-Barreiraa et al. applied 2D-DIGE approach to study proteins extractable by reducing agents from two *A. fumigatus* morphotypes: germlings and hyphae, two adhesin proteins enolase and GAPDH were over expressed in mature *A. fumigatus* hyphae (72 h) and both these proteins were also found in mycelium of *A. flavus* (Kubitschek-Barreira et al., 2013; Pechanova et al., 2013).

During growth, various isoforms of SOD like AfSOD1 and AfSOD2 were highly expressed in conidia and also found in hyphal and mycelium (Vödisch et al., 2009; Cagas et al., 2011b). However, MnSOD (AfSod3p) observed to be over expressed (10-fold) in hyphae and mycelia as compared to conidia (Lambou et al., 2010). AfSOD3p is known to be an allergen (Asp f6), that is readily recognized by IgE of ABPA allergic patients (Schwienbacher et al., 2005a). This antigenic characteristic supports the surface localization of MnSOD (AfSod3p). Whereas, AfSOD1 was observed in mycelial stage of three *Aspergillus* species (Vödisch et al., 2009; Lu et al., 2010; Pechanova et al., 2013). Thus, superoxide dismutase was suggested as a putative virulence factor for this fungus. Apart from these, a CipC-like protein, AFUA_5G09330 found as a major hyphal protein and differential expression of CipC-like genes that was reported for several fungal species found that this protein play an important role in the adaptation of fungi to certain environmental conditions as well as have a relevant role in pathogenicity of *A. fumigatus* (Bauer et al., 2010; Kubitschek-Barreira et al., 2013). This protein is also found in mycelia of *A. flavus* (Pechanova et al., 2013). Additionally, Cagas et al. provided a comprehensive data for four stages of early development and evaluated using gel free system of isobaric tagging for relative and absolute quantification to determine the full proteomic profile of the pathogen. The gel free system of isobaric tagging for relative and absolute quantification (iTRAQ) system has the ability to simultaneously analyze eight samples while identifying hundreds of proteins with quantification for each one relative to any other sample. They observed 24 proteins that showed an increase of 2-fold or greater over the time course (Cagas et al., 2011b). The Ran-specific GTPase activating protein, showed increase of 2.1-fold at 4 h, 2.1-fold at 8 h, and 2.5-fold at 16 h and can be a mycelial specific protein. This protein is also reported in mycelia of *A. flavus* (Pechanova et al., 2013).

In case of *A. niger* very few common mycelial proteins were observed, those were not significant to be compared with the other species. Thus, in case of *A. niger*, it should be of great interest to focus on the studies which like to assess the proteins that are both turned on and turned off during its developmental process. Apart from this, while reviewing the data, it was observed that two-dimensional gel electrophoresis has been the standard approach for the past years, but it has the limitations of profiling as only the most abundant proteins are identified and require tedious quantification. Presently, researchers are analyzing samples kinetically from conidia to young hyphae, then to mycelial stage and comparative proteome profiling to those that are increasing or decreasing in response to the antifungal drug. Further, Cagas et al. made an attempt to profile the *A. fumigatus* proteome in response to caspofungin to observe the effect of this drug on proteins at different stages of growth (Cagas et al., 2011a). This generates critical protein data for the identification and evaluation of new biomarkers of active aspergillosis infection and possible new antifungal targets.

## Virulence determinants of *Aspergilli*

The pathogenicity of *Aspergillus* species depends upon various factors such as status of host immune system and type of strain infecting (environmental strain or clinical strain; Paulussen et al., 2017). The combination of these factors contributed to the successful development of *Aspergilli* inside infected hosts. Most important factor is the virulence determinants that contribute for successful invasion into host cells and tissues. Therefore, factors such as proteins, enzymes, secondary metabolite, or toxin that contribute to virulence may be considered as true virulence factors for *Aspergilli* (Latgé, 1999). Significant work has been done to assess the virulence of *A. fumigatus* using mice models. Mice models helped to understand the pathogenicity and virulence characteristics of *Aspergilli* especially of *A. fumigatus* that lead to development of therapeutic or diagnostic molecules (Clemons and Stevens, 2005). There are different molecules that contribute to *Aspergillus* species virulence such as cell wall components, conidial pigments, mycotoxins, and various reactive oxygen species. Among these categories, two of them can be considered as virulence factor likewise mycotoxins and melanin pigment (Rementeria et al., 2005). Mycotoxins or melanin pigment are not required for growth of *Aspergilli* but help them to survive in extreme conditions (Kwon-Chung and Sugui, 2013). Previously it has been reported that the loss of melanin of *A. fumigatus* conidia make it avirulent when tested in BALB/c mice model (Tsai et al., 1998). Among cell wall components such as polysaccharides β (1-3) glucan, and galactomannan (Afm1p & Afm2p) and proteins/enzymes associated to cell wall β (1-3) glucan synthase complex, chitin synthase and α (1-3)-glucan synthase may act as virulence factors and contributed to fungal growth inside infected host (Latgé et al., 2005; Rementeria et al., 2005; Maubon et al., 2006). β (1-3) glucan significantly modulate the immune response of infected host and activates inflammatory mediators (TNF-alpha and leukotrienes; Ishibashi et al., 2004; Thakur and Shankar, 2017b). Previously, Tiwari et al. observed the expression of β (1-3) glucan synthase in germinating conidia of *A. flavus* and thus contributed to the growth *A. flavus* inside host (Tiwari et al., 2016). In another proteomic study, β (1-3) glucan synthase was also observed during germination of *A. terreus* conidia (Thakur and Shankar, 2017a). The other proteins associated to conidia of *Aspergilli* such as hydrophobins (RodAp & RodBp) contributed to rodlet layer formation and proteins involve in conidial pigments synthesis (polyketide synthase, hydroxynaphthalenes reductase) help in the evasion of immune response hosts (Paris et al., 2003; Aimanianda et al., 2009; Thywißen et al., 2011). Also, role of RodA has been shown in conidial hydrophobicity, sporulation and resistance to physical stress (Valsecchi et al., 2018). In proteomic study on early development of *A. fumigatus*, the high expression of hydrophobin protein RodA and Abr2 that involve in melanin synthesis was observed in conidia and their expression decreased in another morphotypes (Cagas et al., 2011b). Further, Guatam et al. observed the downregulation of RodB proteins in *A. fumigatus* upon treatment of Amphotericin B in comparison to untreated condition. This data suggested that the RodB involved in integration of cell wall and fungal growth and help in the invasion of host tissues (Gautam et al., 2008). Furthermore, Suh et al. observed virulence factors in *A. fumigatus* proteome study, the over expression of Mn superoxide dismutase SodB (Allergen), conidial pigment biosynthesis proteins Arp1 and Ayg, endopeptidase and 2-methylcitrate synthase McsA were observed in conidia as compared to other morphotypes (Suh et al., 2012).

Other major contributors to virulence in *Aspergilli* are mycotoxins produced by various *Aspergillus* species, among *A. fumigatus*; gliotoxins significantly contribute to virulence and invasion of host tissues (Spikes et al., 2008). It acts as a cytotoxic agent to macrophages and respiratory epithelium as well as inhibit NADPH oxidase thus affects neutrophils functions (Tsunawaki et al., 2004; Fujimoto et al., 2016). Toxin produced by *A. fumigatus* is diffusible which inhibits the macrophage phagocytosis and cytokine expression(Abad et al., 2010). Other toxins produced by *A. fumigatus* such as ribotoxin, fumagillin, aurasperone, and fumigacin, contribute to invasion in host systems (Kamei and Watanabe, 2005; Fallon et al., 2011). Using recombinant mitogillin (ribotoxin) (Schwienbacher et al., 2005b), anti-mitogillin antibodies has been detected in the sera of invasive aspergillosis patients suggesting their role in pathogenesis.

Aflatoxin is the important secondary metabolite produced by *A. flavus*. It is the potent carcinogenic or toxic products ever present in nature. The consumption of contaminated food crops lead to aflatoxicosis in humans and other mammals (Richard et al., 1984). The experimental studies showed that the aflatoxin can suppress the phagocytosis and intracellular killing by macrophages along with blocking the production of superoxide (Cusumano et al., 1990). *A. terreus* also produced various mycotoxins such as gliotoxins, geodin, patulin, and terretonin which can act as virulence factor for *A. terreus* and may contribute to invasion of host tissues (Samson et al., 2011; Thakur and Shankar, 2017a). Since, there have been limited studies on each of these mycotoxins focusing on their role in pathogenesis or how it affects the host cells, more studies in relation to mycotoxin to the host cells are required.

Determinants of *Aspergilli* virulence are also the siderophores that help to uptake iron from host and help fungus to grow inside infected hosts (Haas, 2014). *A. fumigatus* produces various hydroxamate siderophores which includes ferrichrome C, ferricrocin, and triacetylfusarinine C (TAF). These siderophores help *A. fumigatus* to use transferrin-bound iron and that lead to the growth of this fungi in the presence of serum or in infected hosts (Hissen et al., 2004, 2005). Now it has been considered that proteins that are involved in signaling pathways, could also be required for the pathogenicity or virulence of *Aspergillus* species. The calcineurin/calmodulin signaling pathway emerged as a key signaling pathway involved in pathogenicity of *A. fumigatus*. The knock out study demonstrated that the deletion of gene CalA which encodes calcineurin phosphatase catalytic subunits results in the reduced growth of *A. fumigatus* and also affects its virulence character (Juvvadi et al., 2014). Other signaling pathways that required for growth and pathogenicity of *A. fumigatus* are mitogen activated protein kinases pathways (MAPK) and cAMP pathway. It has been reported that the MAPKs are required for cell wall integrity in *A. fumigatus*. The cell wall integrity signaling in *A. fumigatus* required three kinases likewise Bck1, Mkk2, and MpkA which help to phosphorylate each other. After phosphorylation MpkA move to nucleus and activates regulators of transcriptions. Mutational studies showed that the mutant of these MAPKs do not phosphorylate and lead to the reduced growth of *A. fumigatus* and filament formation (Jain et al., 2011; Valiante et al., 2015). In *in vivo* condition, large and swollen hyphae with decreased tissue invasion have been observed in farnesyltransferase-deficient mutant of *A. fumigatus* (Norton et al., 2017). In addition, attenuated virulence has been observed in the murine model of invasive aspergillosis. Farnesyltransferase is involved in protein localization and signaling for multiple proteins, including Ras GTPases.

In *A. flavus* germinating conidia, Tiwari et al. observed the expression of mitogen activated protein kinase MpkC, protein kinase C, serine threonine protein kinase MARK2. Thus, these protein expressions during germination revealed the cell wall biogenesis and remolding further contributing to the virulence properties in *A. flavus* (Tiwari et al., 2016). In another study on *A. terreus*, Thakur and Shankar observed the expression of rRNA processing protein (CgrA), myosin-1, MpkC, and mitogen activated protein kinase (hog1) which were reported as virulent factors in *A. fumigatus* using knockout studies (Bhabhra et al., 2004; Hohl and Feldmesser, 2007; Renshaw et al., 2016; Thakur and Shankar, 2017a). Further, they have observed the expression of another protein terrelysin which acts as a hemolysin and help pathogen in invasion into the host tissues.

*Aspergillus* species produce various enzymes or proteins that help to establish successful growth in the hostile environment (de Vries and Visser, 2001). The filamentous fungi produce extracellular enzymes that degrade the physical barrier of hosts that are composed of proteins. *A. fumigatus* produces proteases, elastases, and collagenases enzyme that act as virulence factor and help in invasion of host tissues (Dagenais and Keller, 2009; Behnsen et al., 2010). The human lung matrix composed of collagen and elastin fibers (Balestrini and Niklason, 2015). Thus, these secreted enzymes help to destroy these physical barriers and help the pathogen to grow inside lung matrix. Further, secreted proteases by *A. fumigatus* disorganize the cytoskeleton of alveolar epithelial cells, allowing *A. fumigatus* to breach the epithelial cell barrier (Kogan et al., 2004). Furthermore, some of these proteases such as alkaline serine protease and metalloprotease act as allergen and lead to devastating immune response that ultimately damage other organs in infected hosts (Monod et al., 1993). These reports demonstrated that virulence is multifactorial process (Tomee and Kauffman, 2000). Thus, proteolytic enzymes are one of the factors, each with their part contributing toward the pathogenesis. On the other hand, in *A. fumigatus* proteomic study, endoprotease PEP2 was observed from conidial surface which act as allergen (Asif et al., 2006). In addition, Asp fl2 was observed in *A. flavus* during germination conidia (Tiwari et al., 2016), which trigger the allergic response in infected host. These allergens and other proteins may also be investigated for their role in *Aspergillus* virulence in vivo during infection.

## Biosynthesis of secondary metabolite during different morphological stages of *Aspergillus* species

*Aspergillus* species are known to produce secondary metabolites having commercial or medical importance. Secondary metabolites are the natural products produced in *Aspergillus* species in response to environmental conditions and are the key driver to investigate the extent of toxigenicity and virulence against both plant and animal host. In brief, secondary metabolites are involved in survival of the producer in its environment by competitive inhibition of other organisms (Fox and Howlett, 2008), however exact mechanism is not clear. Thus, secondary metabolite biosynthesis has become an important area to study the event of contamination in pre and post harvested food crops and their role in pathogenesis of *Aspergillus* mediated health problems. To understand the sequential events of secondary metabolite production in *Aspergillus* species, proteomic approach has been proven as the milestone, which also uncovered new aspects of fungal system biology. *Aspergillus* species majorly exist in three different types viz. conidia, hyphae, and mycelia. Between conidial and mycelia stage, germ tube stage formation is the critical step (Harris, 2006). Conidia are dormant morphotypes in the environment and require favorable conditions for germination, a key step in *Aspergillus* related pathogenesis and mycotoxin production (Osherov and May, 2001). It is well established that mycotoxin production occurs at mycelia stage of *Aspergillus* species, but it is equally important to know the sequential events of biosynthesis of mycotoxin at early germination stage of *Aspergillus* species. In the event of mycotoxin biosynthesis at early stages of morphogenesis in fungi could provide the new opportunity to improve diagnosis and identify a common target for multiple *Aspergillus* related infections. Also, it is equally important to understand that conidia of *Aspergillus* species require secondary metabolite intermediates to overcome dormant stage or needed for germination. Secondary metabolites also have the potential for evaluation or development of non-invasive diagnostic method for *Aspergillus* related infection (Ozdemir et al., 2016).

*Aspergillus fumigatus* is a predominant species among *Aspergillus* species in clinical samples and known to produce a class of mycotoxin, gliotoxin, which belong to epidithiodioxopiperazines (Bell et al., 1958). It is majorly involved in apoptotic process, inhibition of NF-κB activation and in prevention of angiogenesis (Pardo et al., 2006; Ben-Ami et al., 2009). Kupfahl et al. showed production of gliotoxin during infection in clinical isolate in mice model, which suggests gliotoxin biosynthesis pathway could be a way for development of metabolite based diagnostic marker against aspergillosis (Kupfahl et al., 2008; Spikes et al., 2008). A shotgun proteome approach for *A. fumigatus* mycelia protein from 2D proteome map showed expression of enzymes involved in gliotoxin, pseurotin A, fumitremorgins, and fumagillin biosynthesis cluster. Other Proteins were phosphoglycerate kinase (PgaK), FAD binding monoxygenase, mitochondrial enoyl reductase, lysophospholipase 3, glutathione S transferase (GliG), thioredoxin reductase (GliT), phytanol-CoA dioxygenase family protein, polyketide synthase, DltD N-terminal domain, O methyltransferase, steroid monooxygenase, phytanol CoA-dioxygenase, acetate CoA ligase, cytochrome P450 oxidoreductase (OrdA), α, β-hydrolase, hybrid PKS NRPS enzyme, methyltransferase (SirN) (Owens et al., 2014). Recently Steve et al. demonstrated full proteome profile at early germinating stages of *A. fumigatus* (0, 4, 8, and 16 h), which did not show expression of any protein/enzyme related to biosynthesis of mycotoxin. These observations may suggest that the event of biosynthesis of mycotoxin starts at the mycelia (fully developed) stage of *A. fumigatus* (Cagas et al., 2011b). However, Moo et al., showed the proteome profile of *A. fumigatus* conidia which resulted into expression of methyltransferase (SirN) and AFUA_8G00550 protein encoded by pseurotin A biosynthesis gene cluster. These findings could be further explored for the diagnostic markers in conidial stages of *A. fumigatus* (Suh et al., 2012). *A. fumigatus* is known to form biofilm from hyphae which includes melanin, polysaccharide and DNA and is involved in decreasing susceptibility toward antifungals (Kaur and Singh, 2014). Burns et al. using proteomic approaches showed the expression of glutathione S-transferase (GliG) and gliotoxin oxidoreductase (GliT) involved in gliotoxin biosynthesis pathway, further confirmed by HPLC analysis (Bruns et al., 2010), which can be further explored as a marker against the virulence of *A. fumigatus*. Also, *A. fumigatus* in response to hypoxia condition (host environment) showed expression of O-methyltransferase (GliM) and pseurotin A (Vodisch et al., 2011) and Gliotoxin oxidoreductase (GliT), MFS transporter (GliA) when provided with exogenous gliotoxin (Owens et al., 2015). These findings provided a global view of activation of gliotoxin pathways and biological processes active under a set of different morphological conditions and stresses for identification of novel cluster products.

*Aspergillus flavus*, a filamentous fungi, known to produce a naturally occurring polyketide, aflatoxin (B1 and B2), which has carcinogenic effects on humans and animals (Giray et al., 2007; Reddy et al., 2011). Approximately 4.5 billion people in developing nations are at the risk of unchecked amount of aflatoxin which results in acute aflatoxicosis (O'brian et al., 2007; Mwalwayo and Thole, 2016). The World Health Organization advised that even low doses with dietary exposure to aflatoxin is a major risk and can lead to hepatocellular carcinoma (Magnussen and Parsi, 2013). *A. flavus* proteomic data at different stages (germinating conidia, mycelia) has shown a visible picture of the expression of proteins during morphogenesis of fungi. Protein analysis by using 2DE and MALDI-TOF-MS/MS of mycelia stage of *A. flavus* showed the active involvement of aflatoxin biosynthesis pathway. Proteins like O-methyltransferase A (OmtA) and AflK/vbs/VERB synthase, ver-1, norA, ver-1, aflatoxin B1-aldehyde reductase GliO-like were expressed (Pechanova et al., 2013). However, a nLC-Q-TOF mass spectrometric analysis of *A. flavus* at germ tube stage (7 h) showed the expression of AflR, AflN, versicolorin B-desaturase, sterigmatocystin-8-O-methyltransferase, oxidoreductase, norsolorinic acid reductase, averufin oxidase-A. It suggests that *A. flavus* if provided with favorable conditions, starts the biosynthesis of aflatoxin at early germination stages (Tiwari et al., 2016). In another study where *A. flavus* was provided with a favorable substrate (corn flour), expression of important enzymes involved in aflatoxin biosynthesis pathway, such as AflR (regulatory protein), nonribosomal peptide synthetase 10, subunit α and β of fatty acid synthase, sterigmatocystin biosynthesis P450 monooxygenase, polyketide synthase (PksA), noranthrone synthase, noranthrone monooxygenase, acetyl-CoA synthatase, NOR reductase, P450 monoxygenase, averantin hydrolase, oxidase, esterase, desaturase, O-methyltransferase-A, alcohol dehydrogenase was identified. These findings suggest that when substrate is added, aflatoxin biosynthesis gets activated and is required for the growth of fungus. However, no data on secondary metabolite biosynthesis at conidial stage of *A. flavus* is available. Additionally, *A. flavus* grown on corn flour when treated with an inhibitor (quercetin) at germinating stage, showed expression of only few enzymes such as, polyketide synthase AflC/PksA/PksL1, fatty acid synthase subunit-α and β, oxidoreductase, alcohol dehydrogenase, non ribosomal peptide synthase (Tiwari and Shankar, 2018). These findings showed the inhibition of majority of proteins, which suggests that compounds having inhibitory activity, may target the biosynthesis of aflatoxin, which needs to be explored further.

Another opportunistic species, *A. terreus* is emerging as a fatal pathogen in immunocompromised patients (Hachem et al., 2014). Recently, analysis of germinating stage of *A. terreus* conidia through nLC-Q-TOF showed expression of enzymes involved in biosynthesis of geodin and terretonin such as terpene cyclase (Trt1), FAD binding monoxygenase (Trt3), isomerase (Trt4), methyltransferase (Trt5), cytochrome P450 monoxygenase (Trt6), dioxygenase (Trt7) and dehydrogenase (Trt9) for terretonin pathway and methyltransferase (GedA), ACP thioestrase (GedB), atrochrysone carboxylic acid synthase (GedC), glutathione S-transferase (GedE), anthrone oxidase (GedH), decarboxylase (GedI), dihydrogeodin oxidase (GedJ), oxidase (GedK), and sulochrine halogenase (GedL) in geodin biosynthesis pathway. However, in conidial stage of *A. terreus* only few proteins/enzymes were found to be expressed such as terpene cyclase (Trt1) for terretonin, non-reducing Pks (TerA) for other secondary metabolite and atrochrysone carboxylic acid synthase for geodin biosynthesis (Thakur and Shankar, 2017a). These observations showed the activation of mycotoxin biosynthesis pathway during germinating stage of *A. terreus*, which may provide an insight into the mechanism of secondary metabolite biosynthesis (Koo et al., 2014) and can be further studied as better non-invasive diagnostic marker or vaccine candidate at early stage of infection.

*Asergillus niger* is known to produce ochratoxins (mainly ochratoxin A) and fumonisins (mainly fumonisins B2) which is a carcinogenic compound and affects the kidney functions in humans (Abarca et al., 1994; Frisvad et al., 2007; Hadjeba-Medjdoub et al., 2012). Two-D electrophoresis and MALDI-TOF proteome analysis of *A. niger* grown on lactate added starch showed the higher expression of intracellular acetyl-CoA (fumonisin precursor) and NADPH, which suggested the regulation of fumonisin by acetyl-CoA in mycelia stage (Sørensen et al., 2009). The studies on secondary metabolism biosynthesis pathway at different morphological stages of *A. niger* need to be further investigated. The list of proteins involved in mycotoxins biosynthesis pathway expressed in different morphological stages /development in *Aspergillus* species are given in Table 3.

**Table 3:** Summarization of expressed enzymes/proteins involved in mycotoxin biosynthesis.

***Aspergillus* species**	**Mycotoxin produced**	**Proteins/Enzymes identified**		**Development stage (time)**	**References**
*Aspergillus flavus*	Aflatoxin B1, B2	Sabouraud dextrose media	AflR, AflN, Versicolorin B desaturase, Sterigmatocystin-8-O-methyltransferase, O methyl sterigmatocystin oxidoreductase, Norsolorinic acid reductase, Averufin oxidase A	Germ tube stage (7 h)	Pechanova et al., 2013; Tiwari et al., 2016
	Aflatoxin B1, B2	Favorable substrate (corn flour)	AflR, Nonribosomal peptide synthetase 10, α and β- fatty acid synthase, Sterigmatocystin biosynthesis P450 monooxygenase, PksA, Noranthrone synthase, Noranthrone monooxygenase, Acetyl CoA synthatase, NOR reductase, P450 monoxygenase, Averantin hydrolase, oxidase, esterase, Desaturase, O methyltransferase A, alcohol dehydrogenase	Germ tube stage (7 h)	Tiwari and Shankar, 2018
	Aflatoxin B1, B2	Inhibitor (Quercetin)	Polyketide synthase AflC/ PksA/ PksL1, Fatty acid synthase subunit-α and β, oxidoreductase, Alcohol dehydrogenase, Non ribosomal peptid synthase	Germ tube stage (7 h)	Tiwari and Shankar, 2018
	Aflatoxin B1, B2	Potatoes dextrose media	O-methyltransferase A (omtA) and AflK/ vbs/ VERB synthase, ver-1, norA, ver-1, aflatoxin B1-aldehyde reductase GliO	Mycelia	Pechanova et al., 2013
*Aspergillus fumigatus*	Gliotoxin	Minimum Essential Medium *(*MEM) with 5% Fetal calf serum	Glutathione S-transferase (GliG), Gliotoxin oxidoreductase (GliT)	Mycelia (Biofilm)	Bruns et al., 2010
	Gliotoxin	Provided with exogenous gliotoxin	Gliotoxin oxidoreductase (GliT), MFS transporter (GliA)	Hyphae	Owens et al., 2014
	Gliotoxin	Hypoxia	O-methyltransferase (GliM)	Mycelia	Vodisch et al., 2011
	Gliotoxin, Pseurotin A, Fumitremorgins and Fumagillin	*Aspergillus* minimal media, Czapek-Dox media	PgaK, FAD binding monoxygenase, Mitochondrial enoyl reductase, lysophospholipase 3, GliG, GliT, phytanol-CoA dioxygenase family protein, polyketide synthase, DltD N-terminal domain, O methyltransferase, steroid monooxygenase, phytanol CoA-dioxygenase, acttate CoA ligase, OrdA, α, β-hydrolase, hybrid PKS NRPS enzyme, SirN	Mycelia	
	Pseurotin A	Glucose minimal media	Methyltransferase (SirN) and AFUA_8G00550 protein	Conidia	Suh et al., 2012
*Aspergillus niger*	Fumonisins B2 and B4	Czapek Yeast Autolysate agar with starch	Acetyl-CoA, NADPH	Mycelia	Sørensen et al., 2009
*Aspergillus terrus*	Terretonin	Trt1		Conidia	Thakur and Shankar, 2017a
		^*^ Trt1, Trt3, Trt5, Trt6, Trt7, Trt9,Trt 14		Germinating conidia stage	
	Geodin	Atrochrysone carboxylic acid synthase,		Conidia	Thakur and Shankar, 2017a
		^*^ GedA, GedB, GedC, GedE,GedH, GedI, GedF, GedJ, GedK, GedL		Germinating conidia stage	

* *Trt1, terpene cyclase, Trt3, FAD- binding monooxygenase; Trt5, Methyltransferase; Trt6, Cytochrome P450 monooxygenase; Trt7, Dioxygenase; Trt9, Dehydrogenase; Trt14, isomerase*.

* *GedA, O-methyltransferase; GedB, Atrochrysone carboxyl ACP thioesterase; GedC, Atrochrysone carboxylic acid synthase; GedE, Glutathione S-transferase-like protein; GedF, Monooxygenase; GedH; Anthrone oxygenase, GedI, Decarboxylase; GedJ, Dihydrogeodin oxidase; GedK, Questin oxidase; GedL, Sulochrin halogenase*.

## Conclusion and future aspects

In this review, we have provided the list of proteins/enzymes required during different development stages of *Aspergilli*. Signaling cascades that are involved during the transition of morphotypes are MAPK, cAMP, and calmodulin/calceneurin pathway suggesting crosstalk between signaling pathways during morphogenesis. The key morphotypes are dormant conidia, swelled conidia, germling conidia (germinating conidia), hyphae, and mycelia. Availability of water and nutrients allow conidia to shift from fermentive to aerobic repiratory metabolism to grow isotropically to exit the conidial dormancy. Using proteome analysis of single cell or particular cell types of *Aspergillus* species may further improve our understanding on growth and development of *Aspergillus* morphotypes or their transition. Further, to assess the dynamics of morphogenesis, studies on relationship between transcripts and protein abundance during the develomental stages or in response to drugs/stress are gaining importance. Furthermore, dual transcriptome or proteome analysis of *Aspergillus* morphotypes with host immune cells or in mice model may provide advantage to reveal the molecular events during pathogenesis (Shankar et al., 2018). RNA-seq and proteome analysis could be handful in such studies to delineate the unknown mechanisms. This kind of approaches with addition of advanced molecular techniques may reveal the changes in gene product expression during germination and early development, thus may contribute to the understanding of this fungal spp. Overall, fungal development, antifungal drug discovery, biomarker assessment as well as *Aspergillus* pathogenesis needs effort to discover novel biomarkers for accurate diagnosis, as well as for new therapeutic agents.

## Author contributions

JS conceived and designed the review. JS, ST, and RT analyzed the data. JS and PV contributed reagents, materials, analysis tools. JS, ST, SS, MG, SH, RT, and PV contributed to the writing of the manuscript.

### Conflict of interest statement

The authors declare that the research was conducted in the absence of any commercial or financial relationships that could be construed as a potential conflict of interest.
